# Conditional local recurrence risk: the effect of event-free years in different subtypes of breast cancer

**DOI:** 10.1007/s10549-020-06040-3

**Published:** 2021-03-10

**Authors:** M. Moossdorff, Marissa L. G. Vane, T. J. A. van Nijnatten, M. C. van Maaren, B. Goorts, E. M. Heuts, L. J. A. Strobbe, M. L. Smidt

**Affiliations:** 1 Department of Surgical Oncology, Maastricht University Medical Centre, P.O. Box 5800, 6202 AZ Maastricht, The Netherlands; 2 GROW - School for Oncology and Developmental Biology, Maastricht University Medical Centre, Maastricht, The Netherlands; 3 Department of Radiology and Nuclear Medicine, Maastricht University Medical Centre, Maastricht, The Netherlands; 4 Department of Research and Development, Netherlands Comprehensive Cancer Organisation (IKNL), Utrecht, The Netherlands; 5 Department of Health, Technology and Services Research, Technical Medical Centre, University of Twente, Enschede, The Netherlands; 6 Department of Surgical Oncology, Canisius Wilhelmina Ziekenhuis, Nijmegen, The Netherlands

**Keywords:** Breast cancer, Conditional survival, Local recurrence, Breast cancer subtypes

## Abstract

**Background:**

After breast cancer treatment, follow-up consists of physical examination and mammography for at least 5 years, to detect local and regional recurrence. The risk of recurrence may decrease after event-free time. This study aims to determine the risk of local recurrence (LR) as a first event until 5 years after diagnosis, conditional on being event-free for 1, 2, 3 and 4 years.

**Methods:**

From the Netherlands Cancer Registry, all M0 breast cancers diagnosed between 2005 and 2008 were included. LR risk was calculated with Kaplan–Meier analysis, overall and for different subtypes. Conditional LR (assuming *x* event-free years) was determined by selecting event-free patients at *x* years, and calculating their LR risk within 5 years after diagnosis.

**Results:**

Five-year follow-up was available for 34,453 patients. Overall, five-year LR as a first event occurred in 3.0%. This risk varied for different subtypes and was highest for triple negative (6.8%) and lowest for ER+PR+Her2− (2.2%) tumors. After 1, 2, 3 and 4 event-free years, the average risk of LR before 5 years after diagnosis decreased from 3.0 to 2.4, 1.6, 1.0, and 0.6%. The risk decreased in all subtypes, the effect was most pronounced in subtypes with the highest baseline risk (ER−Her2+ and triple negative breast cancer). After three event-free years, LR risk in the next 2 years was 1% or less in all subtypes except triple negative (1.6%).

**Conclusion:**

The risk of 5-year LR as a first event was low and decreased with the number of event-free years. After three event-free years, the overall risk was 1%. This is reassuring to patients and also suggests that follow-up beyond 3 years may produce low yield of LR, both for individual patients and studies using LR as primary outcome. This can be used as a starting point to tailor follow-up to individual needs.

## Introduction

Outcomes such as local recurrence (LR) are usually expressed as five or ten-year probability from the time of breast cancer diagnosis. However, as time progresses and a patient remains event-free, this initial estimate of LR (or other outcomes) may have improved. Event-free time is usually not considered as a prognostic factor. An estimate of prognosis that takes the recurrence-free interval into account is called conditional survival or recurrence.

Earlier publications have addressed conditional overall and disease-free survival in breast cancer patients, however mostly without focus on LR [1–3]. Furthermore, these studies were based on older cohorts that differed from current breast cancer patients in several ways: worse baseline prognosis, diagnosis in a time period when breast cancer screening was unavailable, incomplete information on intrinsic subtypes including Her2 status, incomplete use of modern (taxane-based) chemotherapy regimens, and incomplete use of trastuzumab for Her2 overexpressing tumors.

The advantage of calculating conditional LR risks is that individual patients can receive more tailored information about their prognosis, which could be reassuring. Furthermore, this information can also help to determine the optimal follow-up time, both for everyday practice and clinical research. After treatment for breast cancer, follow-up consists of physical examination and mammography for at least 5 years. Thereafter, recommendations vary with regard to frequency, duration, and required investigations. One of the goals of follow-up is to detect possible local and regional recurrences [4–7]. Information on conditional LR risk may be used to tailor follow-up to individual needs. Although extended follow-up may be desirable for other goals such as monitoring endocrine therapy and reassurance, a low chance of events may be a reason to shorten follow-up in specific cases. Safely tailoring follow-up to individual patients could improve quality of care by reducing the number of hospital visits and stress. It can also save health care costs, and may also decrease the required time and financial resources for clinical trials if follow-up can be shortened. In order to preserve quality of care, we need to explore which patients may be eligible for this approach.

Earlier studies on conditional overall and disease-free survival demonstrated the greatest improvement of prognosis (in other words: greatest reduction of the chance of recurrence and death) for patients with the worst prognosis at baseline, which is in line with conditional survival studies for other types of cancer [8–11]. As we hypothesize this may also be the case for LR risk in breast cancer, the role of biologic subtype as prognostic factor may be of interest, in addition to traditional prognostic factors such as tumor size and nodal status. Different subtypes show different patterns of recurrence [12]. It is plausible that the prognostic differences between subtypes depend, among others, on contemporary chemotherapy and trastuzumab. Knowing the effect of event-free years on LR risk in different subtypes could allow tailoring of follow-up, both for clinical practice and trials using LR as an endpoint.

This study aims to determine the risk of LR as a first event within 5 years after diagnosis, conditional on having no breast cancer event for 1, 2, 3 and 4 years. The results will be presented separately for ER+PR+Her2−, ER+PR−Her2−, ER+Her2+, ER−Her2+, and triple negative tumors.

## Methods

### Data collection

The Netherlands Cancer Registry (NCR) collects data on all newly diagnosed cancer patients in all hospitals in the Netherlands from 1989 onward. For the years 2005–2008, both 5-year follow-up on recurrences and information on Her2 status and treatment with trastuzumab are available. Trained data managers of the Netherlands Comprehensive Cancer Organisation (IKNL) obtain data on patient-, tumor- and treatment-related characteristics prospectively from patients’ records. Tumor topography and morphology were coded according to the International Classification of Diseases for Oncology (ICD-O, 3rd edition [13]), and staging was coded according to the tumor, node and metastasis (TNM) classification system (AJCC/UICC, 6th edition [14]). For a period of 5 years after diagnosis, the first breast cancer event was registered (LR, new primary ipsilateral breast cancer, contralateral breast cancer, regional recurrence, or distant recurrence).

### Included patients

From the NCR database, all new invasive epithelial breast cancers diagnosed between 2005 and 2008, of which 5-year follow-up was complete, were included. Patients with distant metastasis at (or within 91 days of) diagnosis were excluded.

### Treatment according to guideline

Patients were treated according to the Dutch national breast cancer guideline of 2005 [15]. Local treatment consisted of breast conserving therapy (lumpectomy and whole breast irradiation) or mastectomy. Post-mastectomy chest wall irradiation was recommended for positive margins, involvement of the pectoralis muscle or skin (T4 tumors), and was considered individually for pT3 tumors. Locoregional radiation was performed for ≥ pN2 or involvement of upper medial axillary nodes. Recommended dose was 45–50 Gy in 5 weeks, or 60–70 Gy in 6 or 7 weeks in case of residual tumor. Lymph node involvement was assessed with sentinel lymph node biopsy (SLNB) for clinically node negative patients according to physical examination and biopsy/fine needle aspiration. Axillary ultrasound was common but not mandatory. Contraindications for SLNB at that time were multiple tumors, > T2, and previous axillary surgery. If SLNB was contraindicated, or if positive lymph nodes were identified either preoperatively or by SLNB, an axillary lymph node dissection (ALND) was performed.

The indication for systemic treatment depended on nodal involvement, tumor size, grade, receptor status, and age. In N+ breast cancer, endocrine therapy was recommended for all patients with ER+ and/or PR+ tumors. Chemotherapy was advised for N+ breast cancer in all premenopausal women and in women < 70 years old with ER- and PR- tumors. In postmenopausal women aged 50–59 with ER+PR+ and N+ tumors, chemotherapy was considered if patients were in good physical condition, and in women aged 60–69 only if four or more of nodes were involved.

For N0 breast cancer, systemic therapy (both chemotherapy and endocrine therapy for ER+ or PR+ tumors and chemotherapy for ER−PR− tumors) was considered for patients ≤ 35 years (except grade I tumors ≤ 1 cm), and for patients > 35 years with tumors ≥ 3 cm, or ≥ 1 cm and grade III, or ≥ 2 cm and grade II. Standard chemotherapy consisted of five courses of FEC (fluorouracil/epirubicin/cyclophosphamide) or six courses of TAC (docetaxel/doxorubicin/cyclophosphamide). If chemotherapy was indicated for a Her2 overexpressing tumor, patients were treated with trastuzumab for one year after chemotherapy.

Endocrine therapy consisted of tamoxifen for 5 years for premenopausal women, optionally including LHRH agonist if not postmenopausal after chemotherapy. For postmenopausal women, either an aromatase inhibitor was given for 5 years, or tamoxifen for 2 years, followed by an aromatase inhibitor.

### Pathology and approximate subtypes

Five subtypes of breast cancer were distinguished, namely ER+PR+Her2−, ER+PR−Her2−, ER+Her2+, ER−Her2+, and triple negative tumours. Tumours were considered ER+ and PR+ then, if more than 10% of tumour cells showed nuclear staining on immunohistochemistry (IHC). Her2 status was evaluated with at least IHC, in which 3+ was considered positive (> 10% of cells with strong intensity circumferential membrane staining) and 0 and 1+ were considered negative (< 10% circumferential membrane staining, or > 10% with weak intensity membrane staining). In case of a 2+ IHC score (> 10% circumferential membrane staining with moderate intensity), fluorescence in situ hybridization (FISH) was mandatory in addition to IHC. If FISH was used, the result of FISH overruled the result of IHC.

### Endpoints

The primary endpoint was (conditional) LR as a first event within 5 years after diagnosis. LR was defined as any invasive breast cancer in the ipsilateral breast (including skin, biopsy tract and surgical scar) or on the ipsilateral thoracic wall including the mastectomy scar, i.e. both LR and new primary ipsilateral breast cancer were counted as LR [16]. Events between 0 and 91 days after diagnosis were regarded as synchronous with the original tumour. Patients were censored at the date of their first event (see “Data collection” section), at the last date of follow-up, or at the date of death. If another event occurred within 91 days of the first recurrence, this was considered synchronous with the first event, and also counted as a first recurrence.

Statistical analyses were performed using SPSS [IBM Corporation, version 23.0.0.0]. Kaplan–Meier analysis was used to determine five-year LR as a first event, for the overall population and separately for five approximate subtypes of breast cancer. To check whether there was an effect of subtype independent of tumor and treatment characteristics, multivariable Cox regression was performed. Variables that were significantly associated with LR on univariable analysis, as well as those known to influence the risk of LR were included in the multivariable analysis. Missing values were disregarded, not imputed. Conditional LR (assuming *x* event-free years) was determined by selecting patients without an event at *x* years, and calculating the risk of LR within 5 years after diagnosis for this selection.

## Results

### Baseline characteristics

In total, the database contained 34.453 new breast cancers diagnosed between 2005 and 2008, of which five-year follow-up was available. Median age was 59.0 years [range: 20–100]. Of these patients, 15.382 (44.6%) were treated with mastectomy, 19.071 (55.4%) with breast conserving therapy. The majority of tumors were ER+PR+Her2− (51.6%), 11.4% were ER+PR−Her2−, 7.8% were ER+Her2+, 5.5% ER−Her2+, and 10.5% triple negative. Of 4548 (13.2%) tumors, subtype was unknown (Table 1).

**Table 1:** Baseline characteristics

Median age (range)		59.0 [20–100]	Morphology	Ductal	25,833 (75.0%)
pT-stage	T0	240 (0.7%)		Lobular	3753 (10.9%)
	T1	20,759 (60.3%)		Mixed ductal/lobular	2122 (6.1%)
	T2	11,547 (33.5%)		Other	2745 (8.0%)
	T3	1036 (3.0%)	Positive margins	No	32,504 (94.3%)
	T4	343 (1.0%)		Microscopic	1398 (4.1%)
	Tx	528 (1.5%)		Macroscopic	49 (0.1%)
pN-stage	N0	20,884 (60.6%)		Unknown	502 (1.5%)
	N1	9157 (26.6%)	Breast surgery	Mastectomy	15,382 (44.6%)
	N2	2533 (7.3%)		BCT	19,071 (55.4%)
	N3	1403 (4.1%)	Radiation therapy	Yes	23,128 (67.1%)
	Nx	476 (1.4%)		No	11,325 (32.9%)
Grade	1	7449 (21.6%)	Chemotherapy	Yes	13,392 (38.9%)
	2	14,275 (41.5%)		Neoadjuvant^b^	1708 (5.0%)
	3	10,204 (29.6%)		No	21,061 (61.1%)
	Unknown	2525 (7.3%)	Endocrine therapy for ER+ tumors	Yes	15,281/27,628 (55.3%)
				Neoadjuvant	369 (1.1%)
ER	Positive	27,628 (80.2%)	Trastuzumab for Her2+ tumors	Yes	2584/4638 (55.7%)
	Negative	6314 (18.3%)	Trastuzumab for Her2+ tumors receiving chemotherapy^a^	Yes	2560/2926 (87.5%)
	Unknown	511 (1.5%)	Subtype	ER+PR+Her2−	17,770 (51.6%)
PR	Positive	21,750 (63.1%)		ER+PR−Her2−	3930 (11.4%)
	Negative	10,960 (31.8%)		ER+Her2+	2689 (7.8%)
	Unknown	1743 (5.1%)		ER−Her2+	1897 (5.5%)
Her2	Positive	4638 (13.5%)		Triple negative	3619 (10.5%)
	Equivocal	1092 (3.2%)		Unknown	4548 (13.2%)
	Negative	26,693 (77.4%)			
	Unknown	2030 (5.9%)	Total		34,453

*ER* estrogen receptor, *PR* progesterone receptor, *BCT* breast conserving therapy

^a^If a patient with a Her2+ tumor was eligible for chemotherapy, this patient was also eligible for trastuzumab

^b^Included in chemotherapy ‘yes’, percentage of total

### Local recurrence as a first event within 5 years in different subtypes

**Fig. 1:**
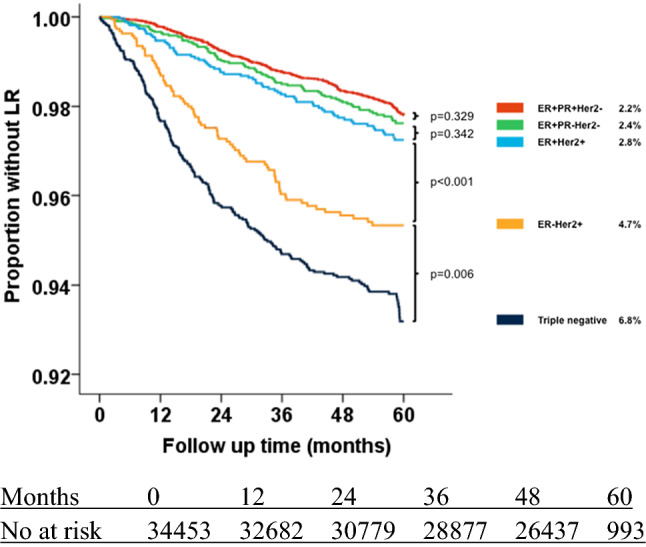
Kaplan–Meier estimator plot of risk of local recurrence as a first event within 5 years after diagnosis in different subtypes of breast cancer

The incidence of LR as a first event within 5 years of diagnosis varied between the subtypes of breast cancer (Table 2, Fig. 1). Incidence was highest in triple negative tumors (6.8%) and lowest in ER+PR+Her2− tumors (2.2%). The difference between the subtypes was significant, except for the difference between ER+PR+Her2− and ER+PR−Her2− (2.2% vs 2.4%, *p* = 0.329); and ER+PR−Her2− and ER+Her2+ (2.4% vs 2.8%, *p* = 0.342). The difference between ER+PR+Her2− (2.2%) and ER+Her2+ (2.8%) was significant (*p* = 0.046).

**Table 2:** Risk of local recurrence as a first event (Kaplan–Meier survival estimates) within 5 years after diagnosis in different subtypes of breast cancer

	*N*	5-year risk of LR at diagnosis (%)	Significance of difference between the Kaplan–Meier curves
All patients	34,453	3.0	
*Approximate subtyp*			
ER+PR+Her2−	17,770	2.2	
			} *p* = 0.329, *χ*^2^ = 0.954
ER+PR−Her2−	3930	2.4	
			} *p* = 0.342^a^, *χ*^2^ = 0.902
ER+Her2+	2689	2.8	
			} *p* < 0.001, *χ*^2^ = 12.599
ER−Her2+	1897	4.7	
			} *p* = 0.006, *χ*^2^ = 7.535
Triple negative	3619	6.8	

*ER* estrogen receptor, *PR* progesterone receptor, *Her2* Her2Neu receptor

Log Rank (Mantel–Cox) was used to compare significance between the Kaplan–Meier curves

^a^ER+Her2+ (2.8%) tumors did not have significantly more LR than ER+PR−Her2− (2.4%), but ER+Her2+ did have significantly more LR than the most favorable subtype ER+PR+Her2− (2.2%), *p* = 0.046, *χ*^2^ = 3.978

### Local recurrence in different subtypes: differences significant on multivariable analysis

Factors that may influence the risk of LR in different subtypes were selected based on known prognostic significance and/or univariable analysis. When corrected for the selected factors using multivariable Cox regression, the difference in LR between ER+PR+Her2− tumors and the other subtypes was still significant (*p* values < 0.05, HRs, CIs and *p* values in Table 3), except for the difference between ER+PR+Her2− versus ER+PR−Her2− which has a HR of 0.954 with *p* = 0.329. Additionally, after correction for these factors, there was no longer a significant difference in LR between patients treated with mastectomy and breast conserving therapy (HR 1.234, 95% CI 0.944–1.614, *p* = 0.124).

**Table 3:** Multivariable Cox regression to assess the impact of breast cancer subtype on 5-year local recurrence as a first event, corrected for confounding factors

	HR	95% CI	*p* value
Subtype vs ER+PR+Her2−	*Ref*		
ER+PR−Her2−	1.134	0.876–1.467	0.341
ER+Her2+	1.535	1.120–2.105	0.008
ER−Her2+	1.525	1.044–2.228	0.029
Triple negative	2.102	1.613–2.740	< 0.001
*Age*			
Per year increase	0.992	0.984–0.999	0.019
*N-stage*			
N+ vs N0	2.152	1.785–2.594	< 0.001
*T-stage*			
T3–4 vs T1–2	2.221	1.581–3.121	< 0.001
*Grade*			
3 vs 1–2	1.530	1.254–1.866	< 0.001
*Breast surgery*			
Mastectomy vs BCT	1.234	0.944–1.614	0.124
*Radiation therapy*			
No vs yes	1.575	1.216–2.039	0.001
*Chemotherapy*			
No vs yes	1.837	1.438–2.346	< 0.001
*Endocrine therapy*			
No vs yes	2.428	1.934–3.049	< 0.001
*Trastuzumab*			
No vs yes	1.656	1.104–2.485	0.015

### The effect of event-free years on the risk of local recurrence within 5 years

For each subtype, the risk of conditional five-year LR was calculated by selecting patients who were event free (i.e. no local, regional, or distant recurrence, no contralateral breast cancer, and no death) at 12, 24, 36, and 48 months. For each time point and each subtype, the risk of LR within 5 years of diagnosis (the end of regular follow-up) was calculated (Table 3). For the overall group, the risk of developing LR before the end of regular follow-up (5 years) was 3.0%. This risk decreased with event-free years, to 2.4%, 1.6%, 1.0%, and 0.6% after one, two, three and four event-free years (Table 4). This decrease in risk was seen in all subtypes, and was proportionally largest in the subtypes with the highest baseline risk (triple negative and ER−Her2+ tumors). After three event-free years, the risk of developing LR before the end of regular follow-up (5 years) was 1% or less in all subtypes but triple negative tumors (Table 4).

**Table 4:** Impact of a number of event-free years on the risk of local recurrence as a first event within 5 years after diagnosis in subtypes of breast cancer

	*N*	Risk of LR at diagnosis (%)	Risk of LR within 5 years after diagnosis, assuming *x *event-free years—*events/persons at risk (%)*			
			After 1 event-free year	After 2 event-free years	After 3 event-free years	After 4 event-free years
All patients	34,453	3.0	2.4	1.6	1.0	0.6
*Approximate subtypes*						
ER+PR+Her2−	17,770	2.2	2.0	1.5	1.0	0.6
ER+PR−Her2−	3930	2.4	2.0	1.4	0.9	0.5
ER+Her2+	2689	2.8	2.2	1.5	1.0	0.4
ER−Her2+	1897	4.7	3.4	2.0	0.7	0.2
Triple negative	3619	6.8	4.6	2.7	1.6	1.1

*LR* local recurrence, *ER* estrogen receptor, *PR* progesterone receptor

### Percentage of LRs occurring in each year of follow-up

On a group level (e.g. in clinical studies) it is of interest to know which proportion of LRs occurs in which years of follow-up. In ER−Her2+ and triple negative tumors, 62.4% and 69.5% of the total number of events occurred in the first 2 years, whereas 40% would be expected when LRs were distributed equally over 5 years of follow-up (100%/5 years = 20% per year). In the ER+ subtypes, the number of LRs was more equally distributed over the 5 years of follow-up (Table 5).

**Table 5:** Number of local recurrences as a first event within 5 years that occurred in each year of followup

	Total no. of LRs	Number of LRs as a first event within 5 years after diagnosis that occurred in each year of follow-up				
		In 1st year^a^	In 2nd year	In 3rd year	In 4th year	In 5th year
All patients	874 (100%)	203 (23.2%)	238 (27.2%)	186 (21.3%)	127 (14.5%)	120 (13.7%)
*Approximate subtypes*						
ER+PR+Her2−	331 (100%)	39 (11.8%)	89 (26.9%)	77 (23.3%)	65 (19.6%)	61 (18.4%)
ER+PR−Her2−	79 (100%)	13 (16.5%)	23 (29.1%)	18 (22.8%)	13 (16.5%)	12 (15.2%)
ER+Her2+	66 (100%)	14 (21.2%)	18 (27.3%)	12 (18.2%)	12 (18.2%)	10 (15.1%)
ER−Her2+	77 (100%)	24 (31.2%)	24 (31.2%)	19 (24.7%)	7 (9.1%)	3 (3.9%)
Triple negative	203 (100%)	81 (39.9%)	60 (29.6%)	31 (15.3%)	14 (6.9%)	17 (8.4%)

*LR* local recurrence, *ER* estrogen receptor, *PR* progesterone receptor

^a^In 1st year: events within 3 months after initial diagnosis were counted as synchronous to the original tumor, thus, 1st year equals 3 months–1 year after diagnosis

## Discussion

This population-based study of 34.453 breast cancer patients diagnosed between 2005 and 2008 showed that the risk of LR as a first event within 5 years after diagnosis was 3.0%. This risk differed significantly between subtypes, with triple negative tumors being at highest risk with 6.8% and ER+PR+Her2− at the lowest with 2.2%. The difference (ER+PR+Her2− compared to the other types) remained significant when corrected for age, T-status, N-status, grade, type of breast surgery, radiation therapy, chemotherapy, endocrine therapy, and trastuzumab (except ER+PR+Her2− compared to ER+PR−Her2−). With increasing number of event-free years, the risk of having a LR before the end of regular 5-year follow-up decreased. After three event-free years, the risk was 1.0% or less in all subtypes except triple negative breast cancer (1.6%). The decrease in the first 4 years after diagnosis was most pronounced in the higher risk subtypes, namely triple negative (6.8% to 1.1%) and ER−Her2+ (4.7% to 0.2%) tumors.

In clinical practice, this means that a breast cancer patient who has been event-free for 3 years, has a risk of 1% or less developing LR as a first event before the end of regular five year follow-up (unless triple negative, than 1.6%). In a research setting (for instance, in a study using LR as an endpoint) for every 100 event-free patients after 3 years of follow-up, one LR can be expected if follow-up is continued until 5 years. This suggests that although recurrences do occur later in follow-up, three-year results may produce similar results to 5 years, depending on the size of the study.

Our results are in line with publications on breast cancer survival and other cancers, suggesting that improvement with event-free years is greatest for tumors with the worst baseline prognosis [8–11]. The results reflect that ER- (particularly triple negative) tumors show relatively many early LRs (within 2 years), whereas ER+ tumors have a fairly constant rate of LRs throughout the 5 years of follow-up. A study investigating conditional disease-free survival in relation to subtype also showed that ER- tumors conditional DFS improved but suggested that conditional survival decreased for ER+ tumors. This study was limited by a very small number of patients at risk after more than three disease-free years [17].

The strength of this approach is the large, nationwide and comprehensive database, which includes substantial numbers of patients, even of the less common subtypes. Further, this study provides specific percentages of the chance of LR after a number of event-free years. Although the information on conditional LR can be partly deduced from the slope of the Kaplan–Meier curve, these exact percentages help using the information on the declining risk for determining the use of continued follow-up, both in clinical practice and breast cancer research. Limitations of this study are the lack of follow-up beyond 5 years, which would have been useful especially for ER+ tumors, in which late recurrences are known to occur [18]. van Maaren et al. showed that the risk of ten-year LR was lowest for the luminal A subgroup (3.9%) and highest in triple negative disease (5.6%) and decreased with the number of event-free years. After nine event-free years, the risk ranged between 0.5 and 0.8% [19].

Further, in a population that was treated according to a guideline, confounding by severity will occur. This is partially overcome by multivariable analysis. Furthermore, confounding by severity is less important in this analysis compared to other studies, as determining exact estimates of the hazard ratios for treatment and tumor characteristics was not an objective of this study. Presented hazard ratios should be interpreted with caution. Furthermore, due to the inclusion period, tumors were classified according to the 6th edition of the AJCC TNM classification. This is, in terms of primary tumor and LR, the same as the current 7th edition [14]. Finally, in this study, no distinction was made between “true recurrences” and ipsilateral second primary breast cancers, both were counted as local events (consistent with an earlier consensus project [16]). This may lead to a higher estimate of LR when compared to studies that do make this distinction.

These results may be used as a starting point for tailoring follow-up to individual needs, both in clinical practice and for breast cancer research. First, a patient who has been event-free for 3 years may ask about the benefit of continued follow-up visits with physical examination and/or mammography to detect LR. Follow-up visits may have different goals beside detecting LR, including monitoring endocrine therapy and encouraging its use, monitoring and treating other side effects of breast cancer treatment, evaluation of psychosocial concerns, and patient reassurance. However, for some patients, a less than 1% chance of finding a LR may be a reason to discontinue follow-up or tailor it to individual needs. National guidelines may use this information to allow personalized decisions about the duration of follow-up. Different guidelines propose slightly different but similar recommendations for follow-up frequency in the first 5 years, and also differ in their recommendations after 5 years (return to screening program, continued annual mammograms, no recommendations) [4, 5, 7, 20]. Of these guidelines, only the ASCO guideline recommends to consider patient preferences and personal risk, based on age, specific diagnosis, and treatment protocol. None of these guidelines describe which specific patient and tumor characteristics should prompt higher or lower frequency or duration of follow-up. Data on conditional LR in relation to subtype may be used as a starting point for tailoring follow-up to individual patients. An even more personalized risk might be calculated with a nomogram, such as proposed by Witteveen et al. [21], partly on the same population. This model, however, does not incorporate the effect of trastuzumab. Additionally, for breast cancer research using LR as an endpoint, the information on the pattern of LR may be used to determine optimal follow-up time for clinical studies.

In conclusion, in this nationwide database including 34.453 breast cancer patients diagnosed between 2005–2008, the incidence of LR as a first event within 5 years was low overall with 3.0%. The incidence was different between subtypes of breast cancer, ER+PR+Her2− tumors posed the lowest risk and triple negative tumors the highest. The risk of developing a LR within 5 years of diagnosis decreased with event-free years. After 3 years, this risk was 1% or less in all subtypes except triple negative cancers. This improvement in prognosis is reassuring to patients during follow-up. It also suggests that follow-up beyond 3 years may have limited yield when it comes to finding additional LR, both for individual patients and clinical studies using LR as the primary outcome. Although there are many reasons to choose longer follow-up, this may be a starting point to tailor follow-up duration to individual needs and preferences.
